# Effect of a Lung Rest Strategy During Ecmo in a Porcine Acute Lung Injury Model

**DOI:** 10.1186/2197-425X-3-S1-A503

**Published:** 2015-10-01

**Authors:** J Araos, P Cruces, P Tapia, L Alegria, P García, T Salomon, F Rodriguez, M Amthauer, G Castro, B Erranz, D Soto, P Carreño, T Medina, F Damiani, G Bugedo, A Bruhn

**Affiliations:** Pontificia Universidad Catolica de Chile, Departamento de Medicina Intensiva, Santiago, Chile; Universidad Andres Bello, Centro de Investigación de Medicina Veterinaria, Santigo, Chile; Hospital El Carmen de Maipu, Unidad de Pacientes Criticos, Santiago, Chile; Clínica Alemana de Santiago, Unidad de Pacientes Criticos, Santiago, Chile; Universidad del Desarrollo, Centro de Medicina Regenerativa, Santiago, Chile

## Introduction

ECMO is used to treat patients who develop refractory hypoxemia and to provide a more protective ventilation. Several guidelines recommend “lung rest” strategies based on variable ventilatory parameters. However, there is limited evidence to support this strategy.

## Objectives

To compare the effect of a lung rest strategy based on near-apneic ventilation (Vt 1-2 ml/kg, PEEP 10, respiratory rate - RR 5 min) versus conventional (Vt 10ml/kg, PEEP 5, RR 20/min), and standard protective ventilation (Vt 6ml/kg, PEEP 10, RR 20/min).

## Methods

Twenty-four domestic pigs (26-36 kg) were anesthetized, mechanically ventilated (Vt 10 ml/kg, PEEP 5, O2 1.0) and invasively monitored. Six animals were used as Sham. in the other 18 lung injury was induced by saline lavages (30 ml/kg per lavage) performed repeatedly in both supine and prone position until PaO2/FiO2 dropped below 250. They were then subjected to a 2-hour injurious ventilation with PCV, PEEP = 0, Pinsp = 40 cmH2O, RR = 10/min, I:E = 1:1, one hour in prone and the other in supine. After completing lung injury (time 0) animals were connected to a saline primed- MEDOS Hilite ECMO circuit by inserting a AVALON 23F double-lumen cannula through the external jugular vein. Blood flow was set at 60-70% of cardiac output. Animals were randomized into one of the three groups and ventilated according to randomization for the following 24 hours. Respiratory and hemodynamic data were collected at times 0, 3, 6, 12, 18 and 24h. After euthanizing animals at time 24h, tissue samples were extracted from the lungs and injury evaluated and scored by light microscopy. Total lung water content was estimated by the wet-dry weight ratio.

## Results

PaO2 decreased significantly in all groups after injury, but was progressively restored after ECMO start, despite the study group. Mean arterial pressure remained within normal limits throughout the study period, whereas MPAP increased significantly after injury but reached values close to SHAM soon after ECMO initiation. Lung wet-dry weight ratio and histological injury score increased significantly in all study groups compared to SHAM. Although non-significant, there was a trend towards a better histological injury score when Vt was reduced.

## Conclusions

In this preliminary analysis, we found no clear advantage of reducing Vt when applying ECMO to support a double-hit animal model of ARDS in regard to resolution of lung edema or gas exchange. However, further work is required to determine if the non-significant reduction in lung injury observed in the near-apneic strategy may be relevant in providing further protection to the injured lungs supported by ECMO.

## Grant Acknowledgment

CONICYT, Fondecyt 1130428

**Figure 1:**
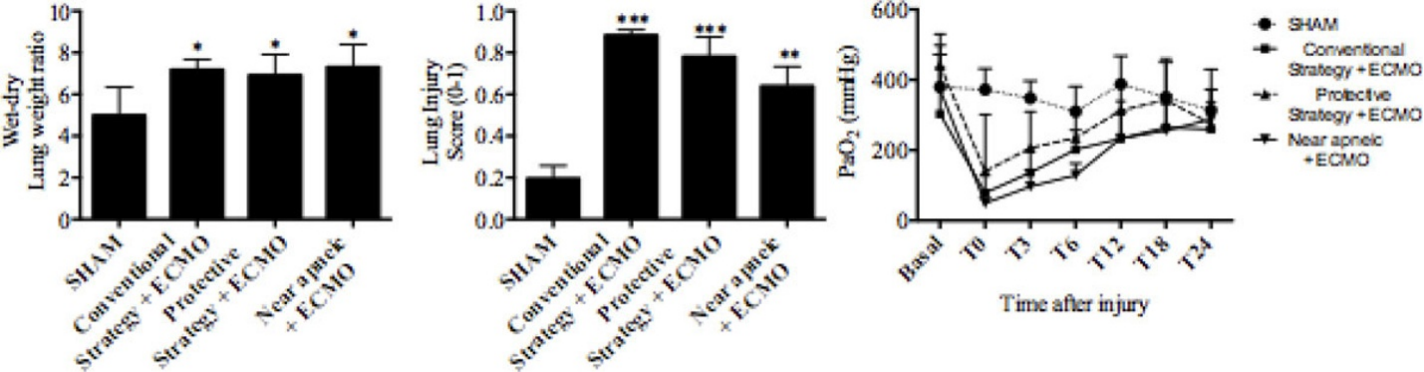
*p < 0.05, **p < 0.01, ***p < 0.001, for lung injury vs SHAM.
